# A C-Terminally Truncated Variant of *Neurospora crassa* VDAC Assembles Into a Partially Functional Form in the Mitochondrial Outer Membrane and Forms Multimers *in vitro*

**DOI:** 10.3389/fphys.2021.739001

**Published:** 2021-09-17

**Authors:** Fraser G. Ferens, William A. T. Summers, Ameet Bharaj, Jörg Stetefeld, Deborah A. Court

**Affiliations:** ^1^Department of Microbiology, University of Manitoba, Winnipeg, MB, Canada; ^2^Department of Laboratory Medicine and Pathobiology, University of Toronto, Toronto, ON, Canada; ^3^Department of Chemistry, University of Manitoba, Winnipeg, MB, Canada

**Keywords:** VDAC, mitochondrial porin, *Neurospora crassa*, dimers, decyl-maltoside

## Abstract

The voltage-dependent anion-selective channel (VDAC) is a porin in the mitochondrial outer membrane (MOM). Unlike bacterial porins, several mitochondrial β-barrels comprise an odd number of β-strands, as is the case for the 19-β-stranded VDAC. Previously, a variant of a VDAC from *Neurospora crassa*, VDAC-ΔC, lacking the predicted 19th β-strand, was found to form gated, anion-selective channels in artificial membranes. *In vivo*, the two C-terminal β-strands (β18 and β19) in VDAC form a β-hairpin necessary for import from the cytoplasm into mitochondria and the β-signal required for assembly in the mitochondrial outer membrane resides in β19. The current study demonstrated that the putative 18-stranded β-barrel formed by VDAC-ΔC can be imported and assembled in the MOM *in vivo* and can also partially rescue the phenotype associated with the deletion of VDAC from a strain of *N. crassa*. Furthermore, when expressed and purified from *Escherichia coli*, VDAC-ΔC can be folded into a β-strand-rich form in decyl-maltoside. Size exclusion chromatography (SEC) alone or combined with multi-angle light scattering (SEC-MALS) and analytical ultracentrifugation revealed that, unlike full-length VDACs, VDAC-ΔC can self-organize into dimers and higher order oligomers in the absence of sterol.

## Introduction

Voltage-dependent anion-selective channels, or mitochondrial porins, reside in the mitochondrial outer membrane and act as general channels that allow the bidirectional flow of metabolites across the membrane [MOM, reviewed in Young et al. (2007)]. Voltage-dependent anion-selective channel pores are modulated by interactions with multiple metabolites, including NADH (Zizi et al., 1994) and proteins such as tubulin (Rostovtseva et al., 2008), thereby contributing to the regulation of cellular metabolism (reviewed in Lemasters and Holmuhamedov, 2006; Rostovtseva and Bezrukov, 2008; Caterino et al., 2017; Shoshan-Barmatz et al., 2017; Magri et al., 2018; De Pinto, 2021). For example, voltage-dependent anion-selective channel (VDAC) interactions with hexokinases (Linden et al., 1982) can inhibit the induction of apoptosis (Azoulay-Zohar et al., 2004). In mammalian systems, VDAC-protein interactions are also associated with a variety of cancers through the promotion of glycolysis and the reduction of apoptosis (reviewed by Mazure, 2017), with VDAC thus being a potential target of therapeutic agents (Shoshan-Barmatz et al., 2019). However, VDAC also participates in other processes (Endo and Sakaue, 2019), such as the import of some proteins into mitochondria (Ellenrieder et al., 2019) and the transport of cholesterol (Liu et al., 2006).

Unlike all characterized bacterial β-barrel proteins, which are composed of an even number of β-strands (Wimley, 2003), mammalian (Bayrhuber et al., 2008; Hiller et al., 2008; Ujwal et al., 2008) and zebra fish (Schredelseker et al., 2014) VDAC, expressed in *Escherichia coli* and folded in detergent, each formed a 19-stranded β-barrel with an N-terminal region that does not contribute to the barrel. Structural predictions for VDACs from multiple sources (Bay et al., 2012) support this model. A comparison of the VDAC isolated in decyl-maltoside (DM)-solubilized mitochondrial membranes of *Neurospora crassa* and the equivalent protein expressed in *E. coli* and folded in the same detergent support the equivalence of the native and recombinant forms (Ferens et al., 2019).

**Figure 1:**
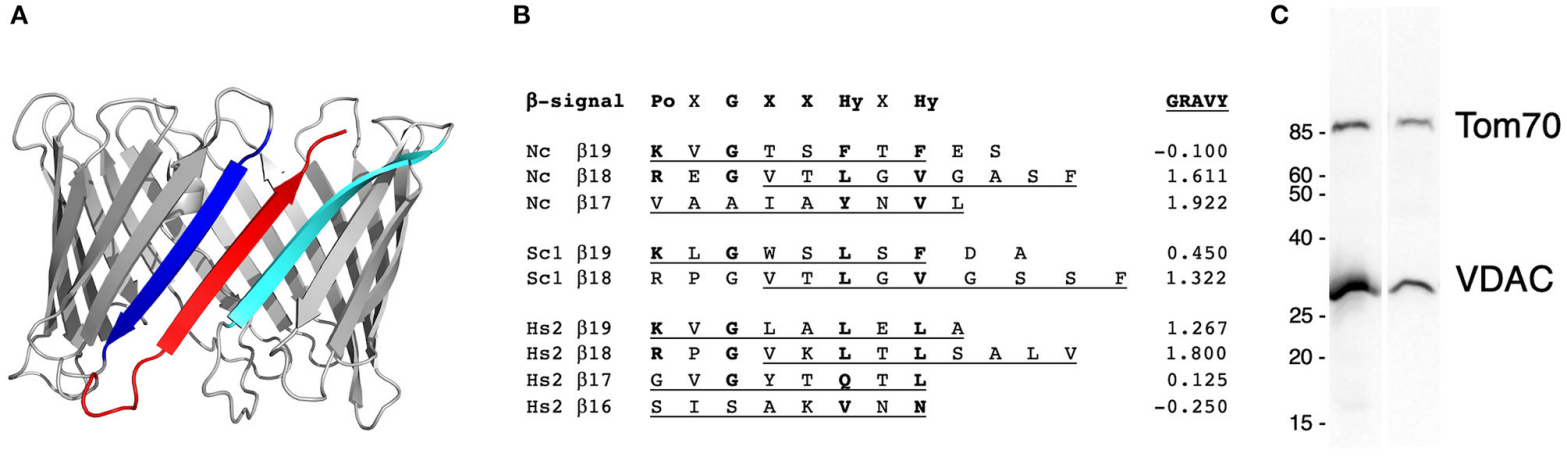
The features and expression of VDAC-ΔC. **(A)** The structural model of *Neurospora crassa* VDAC. The model was generated using Phyre2 (Kelley et al., 2015) with human VDAC1 as the template for the entire sequence (PDB: 2K4T), with a 100% confidence score. β-strands β18 (blue), β19 (red), and β1 (cyan) are highlighted. **(B)** The alignment of the terminal β-strands of *N. crassa* VDAC (Nc, P07144), *Saccharomyces cerevisiae* Por1 (Sc, P04840), and human VDAC 2 (Hs2, P21796, and PDB 2K4T). β-strands predicted by the alignment with human VDAC1 (P21796, 2K4T) in Praline (Simossis and Heringa, 2005) are underlined. Non-X residues matching the β-signal motif (Kutik et al., 2008) are indicated in bold. The grand average of the hydropathicity [GRAVY (Gasteiger, 2006)] score, obtained from https://web.expasy.org/protscale/ is indicated in the right column. Po, polar; X, any residue; G, glycine; Hy, hydrophobic, after (Kutik et al., 2008). **(C)** The Western blot analysis of mitochondria isolated from the wild-type strain (FGSC 9718, left lane) and the strain expressing VDAC-ΔC (right lane). The blot was probed with antibodies against VDAC and Tom70, a component of the protein translocation machinery of the outer membrane (see Supplementary File 1 for details). Size markers on the left are in kDa.

Targeting and assembly of β-barrel proteins into the MOM require two signals in the C-terminal β-strand. A C-terminal hydrophobic β-hairpin is recognized by the receptor Tom20 to initiate import through the TOM complex into the intermembrane space (Jores et al., 2016). Within the terminal β-strand is the β-signal (Kutik et al., 2008) that engages the topogenesis of β-Barrel proteins complex [TOB (Kozjak et al., 2003; Paschen et al., 2003)] or the sorting and assembly (SAM) complex (Wiedemann et al., 2003). This complex, related to the bacterial BAM complex (Gentle et al., 2004), assembles β-barrels in the MOM (reviewed in Hansen and Herrmann, 2019). Thus, the character of the amino acid side chains, rather than a precise sequence, defines the β-signal [Kutik et al., 2008; Imai et al., 2011 (Figure 1B)].

A C-terminal truncation variant of *N. crassa* VDAC, VDAC-ΔC, known as ΔC269-283Por (Popp et al., 1996), lacks the predicted terminal β-strand (β19, Figure 1A). In artificial membranes, it forms anion-selective gated pores similar to wild-type VDACs, but with 75% of the conductance (Popp et al., 1996). Similarly, an 18-β-stranded human hVDAC2 (hV2^18^) lacking β19 forms a pore with about 90% of the conductance of hVDAC2 (Srivastava and Mahalakshmi, 2020). Both 18-stranded molecules form “noisier” channels, but only hV2^18^ shows significantly reduced voltage-dependent gating. Whether this difference is due to an N-terminal His_6_-tag on the *N. crassa* VDAC or the absence of a tag on hV2^18^ remains to be elucidated.

In spite of this pore-forming ability, *N. crassa* VDAC-ΔC synthesized in rabbit reticulocyte lysate is not imported into isolated mitochondria, suggesting that targeting or assembly information is disrupted (Court et al., 1996). In contrast hV2^18^ partially complements the lack of VDAC1 (Δ*POR1*) in *Saccharomyces cerevisiae*, allowing growth on non-fermentable carbon sources (Srivastava and Mahalakshmi, 2020), indicating that it can assemble in mitochondrial membranes.

In the MOM, VDAC exists in various oligomeric arrangements ranging from monomers, dimers, tetramers, and hexamers (Zalk et al., 2005; Geula et al., 2012), as reviewed by Shoshan-Barmatz et al. (2017), to large sheets (Goncalves et al., 2007; Hoogenboom et al., 2007). Hexagonal arrays were observed in negatively stained samples by electron microscopy (EM) upon the depletion of lipids from purified outer membranes (Mannella and Frank, 1984; Mannella et al., 1986) and detected by the atomic force microscopy of both purified outer membranes and spontaneously formed tubular, two-dimensional VDAC crystals (Hoogenboom et al., 2007). The self-association of detergent-solubilized VDAC has been observed in chemically cross-linked samples (Zalk et al., 2005; Malia and Wagner, 2007). In the absence of a cross-linker, *N. crassa* VDAC, expressed in *E. coli* and folded in DM, assembles into higher order structures only in the presence of sterol; models of dimers and of hexamers that resembled those observed by (Mannella et al., 1986) were obtained from a combination of SEC, alone or in combination with small-angle scattering (SEC-SAX) and analytical ultracentrifugation (AUC) (Ferens et al., 2019).

The oligomerization of VDAC regulates multiple processes. In mammalian cells, it is associated with the release of cytochrome *c* and apoptotic regulators (Shoshan-Barmatz et al., 2010), reviewed in (Shoshan-Barmatz et al., 2020). Recently, a role for oligomers of the mouse VDAC1 in the release of mitochondrial DNA during oxidative stress has also been shown (Kim et al., 2019). The N-terminal strand is critical for the oligomerization in human (Shoshan-Barmatz et al., 2010) and mouse (Kim et al., 2019) VDAC1.

In the current study, the functionality of VDAC-ΔC in *N. crassa*, the folded state of VDAC-ΔC, and the propensity of the latter to form oligomers were investigated using the protein expressed in *E. coli* and folded in DM.

## Materials and Methods

The *N. crassa* used in the study was grown as described (Davis and De Serres, 1970). Growth rates were measured in race tubes, containing Vogel's minimal medium at 22–23^o^C. An *N. crassa* strain in which the *POR1* gene is replaced by that for VDAC-ΔC was constructed by replacing the wild-type gene in FGSC 9718 (Colot et al., 2006), with the cDNA for the Δ269-283 Por truncation variant (Popp et al., 1996) downstream of a hygromycin-resistance gene (hygR) for selection (Colot et al., 2006) (Supplementary Tables 1, 2). The “Por”/porin nomenclature for VDAC variants is from earlier studies on the *N. crassa* VDAC (Popp et al., 1996). Following selection on hygromycin, the strain was purified by repeated subculturing to remove untransformed nuclei; the absence of *por1*^+^ nuclei was confirmed by PCR. The measurement of cytochrome spectra was carried out as described (Summers et al., 2012).

The expression, folding, and analysis of WT-VDAC and VDAC-ΔC were carried out as described (Ferens et al., 2019) (see Supplementary File 1). Circular dichroism (CD), size exclusion chromatography (SEC), and AUC experiments were carried out as performed in previous studies (Ferens et al., 2019).

SEC-MALS (SEC with multi-angle light scattering, UV absorbance, and differential refractive index detectors) experiments were conducted under the same conditions as normal SEC runs. Data analysis was done using the software ASTRA by Wyatt Technology (Santa Barbara, CA, USA). After that, VDAC SEC-MALS data were analyzed using the protein-conjugate analysis method in ASTRA (Wyatt, 1993; Andersson et al., 2003; Slotboom et al., 2008). For the description of the method, the ratio of detergent/protein (g/g) in the complex is solved using the equation:

(1) $$\begin{array}{l} \frac{1}{1+\;\delta }{\left(\frac{dn}{dc}\right)}_{Protein}+\frac{\delta }{1+\delta }{\left(\frac{dn}{dc}\right)}_{Detergent}\\ \;=\frac{\Delta RI}{\Delta A_{280}}(\left(\frac{1}{1+\delta }\right)\varepsilon_{0.1\%,\;Protein}+\\ \;\left(\frac{\delta }{1+\;\delta }\right)\varepsilon_{0.1\%,\;Detergent}) \end{array}$$

where δ is the ratio of protein/detergent (g/g), dn/dc is the refractive index increment (0.185 ml/g for protein and 0.146 ml/g for DM), ΔRI is the buffer-subtracted refractive index of the sample, ΔA_280_ is the buffer-subtracted absorbance of the sample at 280 nm, ε_0.1%_ is the extinction coefficient at 280 nm [0.79 ml/(mg^*^cm)] for VDAC, 0.82 ml/(mg^*^cm) for VDAC-ΔC, and 0 ml/(mg^*^cm) for DM]. With δ known, the dn/dc of the protein-detergent complex is determined:

(2) $$\begin{array}{l} {\left(\frac{dn}{dc}\right)}_{Complex}=\;\frac{1}{1+\;\delta }{\left(\frac{dn}{dc}\right)}_{Protein}+\frac{\delta }{1+\delta }{\left(\frac{dn}{dc}\right)}_{Detergent} \end{array}$$

The concentration of the protein-detergent complex (c_Complex_) can now be determined:

(3) $$\begin{array}{l} c_{Complex}=\;{\left(\frac{dn}{dc}\right)}_{complex}\Delta RI \end{array}$$

The Rayleigh ratio (R_θ_), is a measure of light-scattering intensity at angle θ from the incident light:

(4) $$\begin{array}{l} R_{\theta }=\;{\left(\frac{I_{\theta }}{I_{0}}\right)}_{Protein\;solution}-\;{\left(\frac{I_{\theta }}{I_{0}}\right)}_{Buffer} \end{array}$$

where I_θ_ is the intensity of scattered light at angle θ from the incident light and I_0_ is the intensity of the incident light. R_θ_ can be related to the molar mass of the protein-detergent complex (M_w,Complex_) through the following equation (the Zimm method):

(5) $$\begin{array}{l} \frac{K^{*}c_{Complex}}{R_{\theta }}=\\ \left(\frac{16\pi^{2}n^{2}<r_{g}^{2}>}{3\lambda^{2}M_{w,\;Complex}}\right)\sin^{2}\left(\frac{\theta }{2}\right)+\frac{1}{M_{w,\;Complex}}+2A_{2}c \end{array}$$

where λ is the wavelength of the incident light, n is the refractive index of the solute, < ${\text{r}}_{\text{g}}^{2}>$ is the root mean square radius, θ is the angle between the scattered light and incident light, A_2_ is the second virial coefficient, and K^*^ is an optical constant defined by the equation:

(6) $$\begin{array}{l} K^{*}=\;\frac{4\pi^{2}{\left(\frac{dn}{dc}\right)}_{Complex}^{2}n^{2}}{\lambda^{4}N_{A}} \end{array}$$

where N_A_ is Avogadro's number. With data points from multiple light-scattering detectors at different angles around the sample, a plot of (K^*^c)/R_θ_ against sin^2^(θ/2) was fit with a linear function with the y-intercept equal to (1/M_w,Complex_ + 2A_2_c). For dilute SEC experiments, the second term of the y-intercept was assumed to be negligible, allowing for the determination of M_w,Complex_ from the y-intercept:

(7) $$\begin{array}{l} \frac{1}{M_{w}}\approx \frac{1}{M_{w}}+2A_{2}c \end{array}$$

Finally, with M_w,Complex_ and δ, the molar mass of the protein (M_w,Protein_) can be determined:

(8) $$\begin{array}{l} M_{w,\;Complex}=\left(1+\;\delta \right)M_{w,\;Protein} \end{array}$$

## Results

### VDAC-ΔC Partially Complements a ΔPor1 Strain

Although it forms pores in artificial membranes, *in vitro*-synthesized VDAC-ΔC cannot be imported into isolated mitochondria (Court et al., 1996). To determine whether VDAC-ΔC can function *in vivo*, a strain of *N. crassa* was generated, in which the coding sequence for VDAC was replaced by the cDNA-encoding VDAC-ΔC with the deletion of amino acid residues 269–283. The assembly of VDAC-ΔC in mitochondria was confirmed by the Western blotting of isolated mitochondrial proteins (Figure 1C).

To assess the functionality of the C-terminally truncated protein, the growth rate of the VDAC-ΔC strain was determined (Table 1). It grew at about 85% of the wild-type rate, which was faster than the VDAC-less strain, ΔPor1. Cytochrome spectra were used to assess the general functioning of the electron transport chain, as the absence of VDAC is associated with severe cytochrome defects in *N. crassa* (Summers et al., 2012). The levels of cytochromes *b* and *aa*_3_, which function in complexes that include mitochondrially encoded subunits, were intermediate between those of the wild-type and the ΔPor1 strain. In VDAC-ΔC, an increase in cytochrome *c* was observed, which has previously been observed in *N. crassa* with defects in cytochromes *b* and *aa*_3_ (Rifkin and Luck, 1971), including those lacking VDAC (Summers et al., 2012) or expressing an N-terminally truncated VDAC, ΔN-2-12Por (Shuvo et al., 2017), or the internally deleted Δ238-242Por (Ferens et al., 2017). Both of the latter molecules also partially complement the lack of VDAC.

**Table 1:** Genotypes and phenotypes of wild-types and strains expressing voltage-dependent anion-selective channel (VDAC) variants.

**Strain**	**Genotype**	Growth rate at 22 ^**o**^C (cm/24 h)^*^	**Cytochrome concentrations (nmol/mg protein)**	**Strain reference**
FGSC^†^ 9718 (wild-type)	*Δmus51::bar^+^* (a)	7.7 ± 0.4^‡^	*aa_3_:* 0.7 ± 0.1^‡^	Colot et al., 2006
			*b:* 1.3 ± 0.1^‡^	
			*c:* 1.0 ± 0.2^‡^	
ΔPor-1	Δ*por::hph^+^ Δmus51::bar^+^ (a)*	1.6 ± 0.1^‡^	*aa_3_*: 1.1 ± 0.0^‡^	Summers et al., 2012
			*b*: 0.6 ± 0.1^‡^	
			*c*: 1.6 ± 0.1^‡^	
VDAC-ΔC	*porΔC269-283*	6.3 ± 0.3	*aa_3_:* 0.4 ± 0.0	This work
	*hph^+^ Δmus51::bar^+^ (a)*		*b:* 1.0 ± 0.1	
			*c:* 1.7 ± 0.2	

† *FGSC, fungal genetics stock center*.

‡ *Summers et al. (2012)*.

* *n ≥ 3; average and standard deviation shown*.

### Mitochondrial β-Barrel Motif Repeats in β18 and β19

The functioning of VDAC-ΔC indicates that the protein is targeted to and assembled in the MOM. Interestingly, the two motifs required for these processes are partially or fully within β19 (Figure 1B). The targeting of VDAC to the TOM complex requires the hydrophobic β-hairpin formed by β18 and β19 in the wild-type molecules. Given that the outer face of the VDAC pore interacts with the membrane, it is not unexpected that alternative pairs of terminal β-strands can form the requisite hydrophobic β-hairpins (Figure 1B). Similarly, the motif that captures the *N. crassa* β-signal can be found spanning part of the β18 from other organisms (Figure 1B, Supplementary Table 3). A survey of about 480 VDAC sequences (Supplementary Table 3) detected the β-signal in β19 in most sequences, with the exception of those from plants (87/162). A second copy of the β-signal was identified to overlap with β18 in 99% of the mammalian, vertebrate (>99%), yeast (100%), and fungal (98%) sequences sampled (Supplementary Table 3; see Figure 1B). Of the arthropod sequences examined, about 45% contained a putative β-signal in β18 and β19, and 52% of sequences contained three putative β-signals, two of which aligned with those in the other organisms and a third that shared three residues with those of β19 (Supplementary Table 3).

### Recombinantly Expressed VDAC-ΔC Can Be Folded in DM

**Figure 2:**
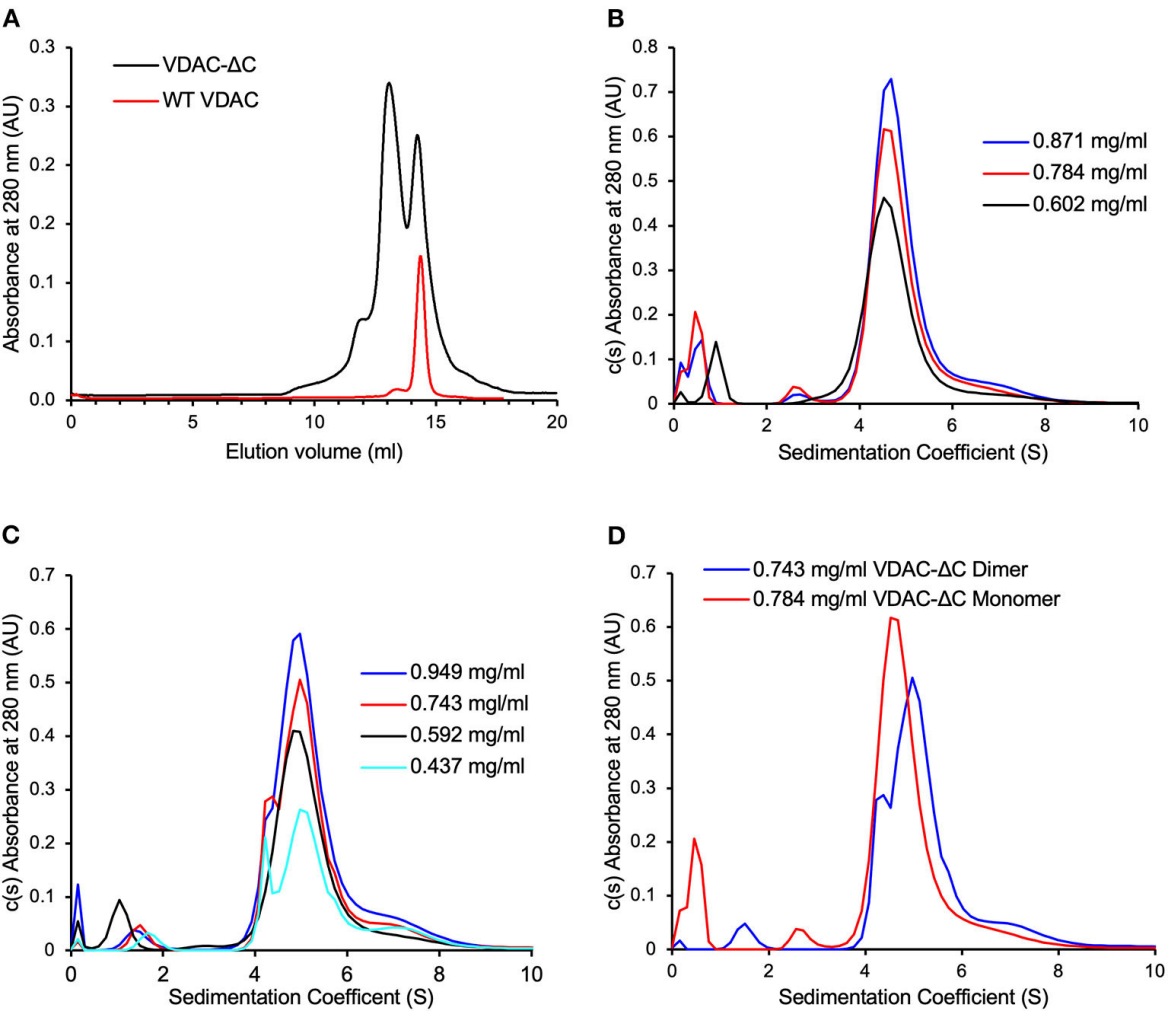
VDAC-ΔC elutes as two major peaks, with the monomers and dimers not returning to equilibration after size exclusion chromatography (SEC) separation. **(A)** WT-VDAC, known to be monomeric, elutes as a single major peak with a similar elution volume to the VDAC-ΔC peak with larger elution volume **(B)** c(s) distribution of the VDAC-ΔC monomer SEC fractions at various VDAC concentrations. **(C)** c(s) distribution of the VDAC-ΔC dimer SEC fractions at various VDAC concentrations. **(D)** Comparison of the VDAC-ΔC monomer and dimer c(s) distributions at similar concentrations, showing differing populations in each sample.

The full-length *N. crassa* VDAC oligomerizes in sterol-containing detergents (Ferens et al., 2019). To explore the ability of the VDAC-ΔC β-barrels to interact with each other, the protein was over-expressed in *E. coli*. It could be purified from inclusion bodies similarly to WT-VDAC and folded in DM using the same methods (Ferens et al., 2019). VDAC-ΔC folds into a predominantly β-strand conformation (Supplementary Figure 1), implying that the protein has formed a large β-sheet or β-barrel. SEC experiments (or Size exclusion chromatography experiments) (Figure 2A) revealed that the VDAC-ΔC in DM elutes in two major peaks, with one at an elution volume like monomeric WT-VDAC (~14.2 ml). The additional peak elutes at about 13 ml, which is very similar to the elution volume of the WT-VDAC dimer that forms in the presence of the cholesterol analog cholesteryl-hemisuccinate (CHS) (Ferens et al., 2019). The deconvolution of the CD spectra of the two more prominent peaks in the elution profile estimated nearly identical secondary structure contributions, suggesting that both species are comprised of similarly folded VDAC molecules (Supplementary Table 4 and Supplementary Figure 1). The VDAC-ΔC species had a ~10% reduction in β-strand content relative to the previously examined WT-VDAC (Supplementary Table 4 and Supplementary Figure 1). Due to the ambiguity in determining the oligomeric states of detergent-solubilized membrane proteins using SEC due to associated detergent molecules, we chose to investigate the two major peaks of DM-folded VDAC-ΔC using SEC-MALS to elucidate the oligomeric states of the two species.

### VDAC-ΔC Folded in DM Is Primarily Comprised of a Mixture of Monomers, Dimers, and Tetramers

**Figure 3:**
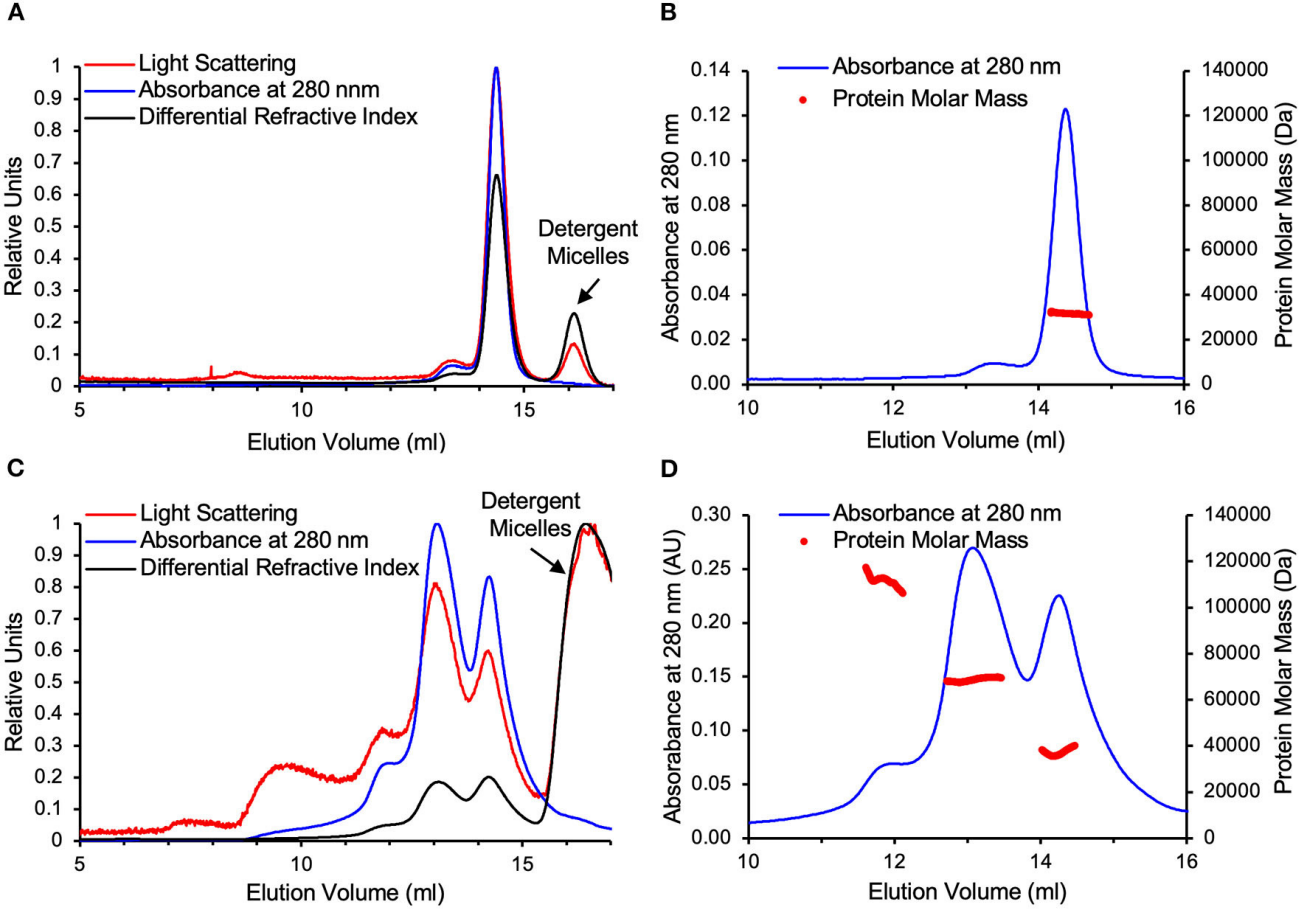
WT-VDAC is monomeric and VDAC-ΔC is composed of monomers and dimers. **(A)** WT-VDAC. SEC with multi-angle light scattering (SEC-MALS) raw data on A relative scale. **(B)** WT-VDAC protein masses across the VDAC peak eluted from the SEC column. **(C)** VDAC-ΔC raw data on a relative scale. **(D)** VDAC-ΔC protein masses across the two major peaks eluted from the SEC column. WT-VDAC and VDAC-ΔC elutions are followed by the elution of excess detergent micelles from the injected samples, which are easily distinguished from VDAC species as they scatter visible light and alter the refractive index but do not absorb UV light. Detergent micelle peaks are indicated in **(A)** and **(C)**.

Both WT-VDAC and VDAC-ΔC were analyzed by SEC-MALS (Figures 3A–D). The elution was monitored by a UV detector, a MALS detector, and a differential refractometer in line with the column, allowing for the deconvolution of the protein molar masses of eluting protein-detergent complexes. The one major peak in the WT-VDAC sample was confirmed to be monomeric with a protein molar mass of 32 ± 1 kDa [theoretical M_w_(tM_w_) 31 kDa], which was in close agreement with previous AUC experiments that analyzed the WT-VDAC in DM (Ferens et al., 2019). VDAC-ΔC was eluted with a small shoulder peak, followed by two major protein-containing peaks. Of the two major peaks, the lower elution volume peak corresponded to the approximate M_w_ of a dimer at 69 ± 1 kDa (tM_w_ 60 kDa), while the higher elution volume peak corresponded to the approximate M_w_ of a monomer at 37 ± 2 kDa (tM_w_ 30 kDa). The small shoulder that preceded the two larger peaks had a protein molar mass of 112 ± 2 kDa and, likely, is a small population of VDAC-ΔC tetramers (tM_w_120 kDa), although the overlapping dimer peak may be interfering with the accurate mass determination of this species. Furthermore, a downward sloping trend in protein mass across this peak was observed (Figure 3D). The deviation of the molar masses determined for the VDAC-ΔC species relative to theoretical values was likely due to the overlapping elution of the three species from the SEC column, which reduced the area of each peak that represented monodispersed species (Figure 3D) (See Supplementary Table 5 for polydispersity values). Regardless, the values determined for the three VDAC-ΔC species were accurate enough to determine the oligomeric states, as the VDAC M_w_ of 30 kDa was far greater than the discrepancies between the theoretical and observed molar masses.

### SEC-Separated VDAC-ΔC Monomers and Dimers Do Not Re-Equilibrate to Identical Populations

As presented above, the SEC elution profiles of VDAC-ΔC contained two major peaks, representing monomers and dimers, which were well defined in the elution profile. This suggested that there was either no equilibrium between the two species or that the rate of transition between monomeric and dimeric forms is very slow. To examine this further, we chose to analyze the SEC-separated monomer and dimer fractions through AUC sedimentation velocity experiments.

Due to the non-ideality caused by weak macromolecular interactions, the overall sedimentation coefficient (S) of noninteracting protein systems usually trends slightly down with increasing protein concentration (Schuck, 2013). In this study, the c(S) distributions of the VDAC-ΔC monomer (Figure 2B) and dimer fractions (Figure 2C) both showed a slight trend of increasing S with increasing VDAC concentrations, suggesting a concentration-dependent association, notably in the dimer sample (Figure 2C), where the resolution between monomer and dimer peaks was clearer. Furthermore, the proportion of monomers and dimers in the distribution seemed to be dependent on the overall VDAC concentration. The distributions of both the monomer and dimer samples contained the same range of species (Figure 2D), suggesting that the equilibration between the oligomeric states of VDAC was present; however, the mixture of species in each sample was clearly enriched in either monomer or dimer (Figure 2D), suggesting that the rate of exchange was very slow as there had not been a complete equilibration of the same population of species in the monomer and dimer samples at similar VDAC concentrations on the timescale of this experiment.

## Discussion

The current work revealed that *N. crassa* VDAC-ΔC can assemble into a functional form *in vivo*. In previous experiments, which used isolated mitochondria and *in vitro-*synthesized VDAC-ΔC, this variant was not imported into protease-resistant conformation mitochondria (Court et al., 1996). Several factors, alone or in combination, could account for this discrepancy. Newly synthesized β-barrel proteins are maintained in import-competent forms by association with Hsp70/Hsp40 family chaperones, which also contribute to their association with the import machinery (Jores et al., 2018). It is possible that either the heterologous (rabbit) chaperones are less effective with the *N. crassa* VDAC or that their concentration was insufficient. Furthermore, even though VDAC-ΔC is at least partially functional, its folded state in the MOM could be more protease-sensitive than that of the wild-type. Finally, the variant of VDAC that was tested in the *in vitro* experiments contained an N-terminal hexahistidinyl-tag (Court et al., 1996), while the variant in *N. crassa* was tag-less, as were the yeast truncation variants (Srivastava and Mahalakshmi, 2020). It is possible that the N-terminal tag interfered with import or generated a more protease-sensitive conformation, even though it did not impair pore function (Popp et al., 1996).

The *in vivo* results obtained with the *N. crassa* VDAC-ΔC agree with those involving hV2^18^ expressed in *S. cerevisiae* (Srivastava and Mahalakshmi, 2020). The latter molecule and those lacking two (hV2^17^) or three (hV2^16^) C-terminal β-strands can partially complement a strain of yeast lacking the primary VDAC, Por1 (Srivastava and Mahalakshmi, 2020). Together, these two studies support the hypothesis that the redundant mitochondrial targeting and assembly information in fungal VDAC is functional. Conversely, the duplication of β19 or of β18 and β19 in hV2^20^ and hV2^21^ led to molecules that more effectively rescue the Δ*POR1* phenotype than do hV2^16^ and hV2^17^ (Srivastava and Mahalakshmi, 2020), presumably because they maintain the native signals in the C-terminal two β-strands.

Biophysical data support the hypothesis that the VDAC expressed in *E. coli* and folded in detergent (recombinant VDAC, VDAC^R^) adopts a similar structure as that isolated from mitochondria (Ferens et al., 2019). The oligomeric state of VDAC^R^ is influenced by the presence of sterol in the detergent micelles; a role for sterol in the biological function of VDAC has been predicted since early evidence of sterol copurifying with VDAC (De Pinto et al., 1989) was found and the more recent identification of sterol-binding regions in the detergent-folded mouse VDAC1 (Cheng et al., 2019). One of the five sterol-binding sites in mVDAC1 involves residue L279 in β19, and, if this binding site is present in *N. crassa* VDAC, it would be disrupted by deletion of β19.

Furthermore, *N. crassa* VDAC folded in DM is monomeric in the absence of sterol, and it is unclear whether the sterols promote a structure more amenable to multimerization or alter the character of the micelles such that they promote dimerization (Ferens et al., 2019). In particular, *N. crassa* VDAC is predicted to fold very similarly to vertebrate VDAC (Bay et al., 2012), with studies on human VDAC1 in detergent (Bayrhuber et al., 2008), rat VDAC1 in cultured cells (Geula et al., 2012), and zebrafish VDAC2 in LDAO (Schredelseker et al., 2014) suggesting a common model in which dimerization involves the interfaces produced by β1, β17, β18, and β19. Therefore, it was unexpected that VDAC-ΔC exited as a mixture of monomers and dimers in the absence of sterol (Figures 2 and 3). This change in the propensity to form oligomers in DM micelles relative to WT-VDAC (Figure 3B) could be due to the creation of a new interaction interface from the assumed interaction of β18 and β1 in antiparallel orientations. This could create a VDAC with a more “bacterial-like” porin architecture. Bacterial porins have been found to form dimers, trimers, and larger oligomers in detergent micelles (Wimley, 2003). Alternatively, the abovementioned sterol-binding site may be interrupted by the VDAC-ΔC deletion. Sterol regulates the WT-VDAC oligomeric state, and the deletion of part of a sterol-binding site may decouple the regulation of the oligomeric state from the binding of sterol molecules. Our AUC analysis suggested that there was some equilibration between VDAC-ΔC species when the SEC-separated monomer and dimer fractions at various concentrations were examined. However, the rate of equilibration was likely to be very slow, which could be due to experimental factors such as the use of detergent micelles as a membrane mimetic system. An obvious complication is the three-dimensional environment of the interaction of the protein-detergent complexes in the solution as compared with the two-dimensional interaction environment of a lipid bilayer. Alternatively, a subset of the VDAC-ΔC molecules in the samples may have been misfolded and, thus, unavailable to form larger species or disassociate into smaller ones. However, the CD spectra of VDAC-ΔC monomer and dimer (Supplementary Figure 1) samples suggest that the VDAC-ΔC molecules in these samples are comprised of a predominantly β-strand fold, which is expected of correctly folded VDAC pores.

Both VDAC and its phylogenetic relatives, Tom40 and Mdm10, exist as 19-stranded β-barrels, which are unlike all known bacterial porins [(Pusnik et al., 2009; Flinner et al., 2013), reviewed in (Bay et al., 2012)]. It has been proposed that, rather than being derived from an ancestral bacterial porin, the progenitor of VDAC and Tom40 was assembled in the last common eukaryotic ancestor from a series of ββ-hairpin units. The resulting 20-stranded barrel was involved in the protein import activity that evolved as genes migrated from early mitochondria to the nucleus (Pereira and Lupas, 2018). The N-terminal helical segment has also been proposed to be derived from the first strand of an ancestral 20-stranded β-barrel as the need for a pore regulatory component arose.

The biological advantage of the 19-β-strand form of VDAC was assessed by generating 16–21 stranded versions of hVDAC2 (Srivastava and Mahalakshmi, 2020). As revealed by enthalpy determinations (ΔH), the 19-β-stranded wild-type form was less stable in bicelles than 18- and 20-stranded variants. Chemical unfolding experiments revealed that the stability of wild-type hVDAC2 was the most sensitive to the environment provided by the lipid head groups. Thus, it was hypothesized that the metastable, 19-stranded molecule allows superior responses to changes in membrane composition, subsequently allowing hVDAC2 to participate in events such as cytochrome *c* release that, in turn, triggers apoptosis. It is also the most compatible with the conductance and gating properties of the molecule (Srivastava and Mahalakshmi, 2020). However, it is unclear whether gating was a selective criterion prior to the divergence of VDAC, Tom40, and Mdm10.

Pore formation and channel gating are among the many functions exhibited by VDAC-ΔC that also are observed in WT-VDAC (Popp et al., 1996; Srivastava and Mahalakshmi, 2020). Herein, it was shown that VDAC-ΔC retains the ability to be imported into mitochondria and inserted into the MOM due to the presence of a second β-signal in β18, which appears to be widely conserved in VDACs from fungi and animals. Furthermore, VDAC-ΔC retains the ability to form dimers and tetramers in detergent micelles, although the conditions under which oligomers form are altered relative to WT-VDAC, which requires the presence of sterol. Thus, VDAC β19 is not essential for pore formation in the MOM; however, it may be necessary for the appropriate self-association of VDAC in response to the surrounding lipid environment.

## Data Availability Statement

The datasets presented in this study can be found in online repositories. The names of the repository/repositories and accession number(s) can be found in the article/Supplementary Material. Other data is available upon request to the corresponding author.

## Author Contributions

WS and AB generated the VDAC-ΔC strain and carried out the biological analysis. FF developed the biophysics pipeline, performed the experimental analyses, and analyzed the data. FF, DC, and JS designed the experiments and prepared the manuscript. All authors contributed to the article and approved the submitted version.

## Funding

This work was supported by the Natural Sciences and Engineering Council of Canada [RGPIN-05930-2016 (DC) and RGPIN-004970-2018 (JS)]. FF was partially supported by the Faculty of Science and the GETS Program, Faculty of Graduate Studies, University of Manitoba (UM). JS is a Canada Research Chair in Structural Biology and Biophysics. We acknowledge the assistance of Richard Adegboyega (UMURA), Emily Ardebol (Faculty of Science URSA), and Nancy Ngyuen (BSc Coop) in collecting and analyzing the VDAC sequences and Max Qually (BSc Honors) for analyzing VDAC-ΔC strains.

## Conflict of Interest

The authors declare that the research was conducted in the absence of any commercial or financial relationships that could be construed as a potential conflict of interest.

## Publisher's Note

All claims expressed in this article are solely those of the authors and do not necessarily represent those of their affiliated organizations, or those of the publisher, the editors and the reviewers. Any product that may be evaluated in this article, or claim that may be made by its manufacturer, is not guaranteed or endorsed by the publisher.
