# Supplementary material for: A C-Terminally Truncated Variant of Neurospora crassa VDAC Assembles Into a Partially Functional Form in the Mitochondrial Outer Membrane and Forms Multimers in vitro
Source: Front Physiol. 2021 Sep 17;12:739001. doi: 10.3389/fphys.2021.739001 (PMC8485043; doi:10.3389/fphys.2021.739001)
Supplement: Supplementary file 1 [file Table_1.docx]

**Supplementary Table 1.** Plasmids used in this study (Summers, W.A.T. 2010. An in vivo approach to elucidating the function of mitochondrial porin by the characterisation of *Neurospora crassa* strains deficient in porin Ph.D. Thesis, University of Manitoba, Winnipeg, MB).

| **Plasmid** | **pRS416 C-terminal His6 VDAC** | **Starting vector: pRS416** |
| --- | --- | --- |
| **Insert** | **Primers** | **Template** |
| +1071 to +2151 *por* (1.3 kb product of cross-stitch PCR) | (1) porTAG_his6 5' and porTAG_his6 3' (2) porTerm 5' and porTerm 3' (3) porTAG_his6 5' and porTerm 3' | (1 & 2) FGSC 9720 genomic DNA  (3) PCR Products of (1 & 2) |
| +2233 *por* downstream UTR (1.2 kb | porTermUTR F and porTermUTR R | FGSC 9720 genomic DNA |
| *hph* | hphFnarI and hphRnarI | pCNS44 |
|  |  |  |
| **Plasmid** | **NcHygΔC** | **Starting Vector:** NcΔC-2 (*Mlu*I digested) |
| **Insert** | **Primers** | **Template** |
| *por* upstream UTR::HygR (2.5 kb) | 5' Prom::pRS416 and NcPprom:cDNA 3' | pRS416 + NcPprom::HygR ATG His6 porin |
|  |  |  |
