# Supplementary material for: A C-Terminally Truncated Variant of Neurospora crassa VDAC Assembles Into a Partially Functional Form in the Mitochondrial Outer Membrane and Forms Multimers in vitro
Source: Front Physiol. 2021 Sep 17;12:739001. doi: 10.3389/fphys.2021.739001 (PMC8485043; doi:10.3389/fphys.2021.739001)
Supplement: Supplementary file 2 [file Table_2.docx]

**Supplementary Table 2.** Primers used in this study ({Summers, 2010 #44}).

| Primer | Sequence (5’-3’) |
| --- | --- |
| porTAG_his6 5' | GTAACGCCAGGGTTTTCCCAGTCACGACGTTGTAAAACGGCGCGCCAAGGGTCTCAAGGC |
| porTAG_his6 3' | CTTCACATGTGTCCTGCGATTACTTACGTACTAGTGGTGGTGGTGGTGGTGGGCAGACTCGAAGGTGAAGCTGGTGCCG |
| porTerm 5' | CGGCACCAGCTTCACCTTCGAGTCTGCCCACCACCACCACCACCACTAGTACGTAAGTAATCGCAGGACACATGTGAAG |
| porTerm 3' | GCCCAAAAAGTGCTCCTTCAATATCATCTTCTGTCGACGGCTTCACTTCATTGCACAGCC |
| porTAG_his6 5' | GTAACGCCAGGGTTTTCCCAGTCACGACGTTGTAAAACGGCGCGCCAAGGGTCTCAAGGC |
| porTerm 3' | GTAACGCCAGGGTTTTCCCAGTCACGACGTTGTAAAACGGCGCGCCAAGGGTCTCAAGGC |
| porTermUTR F | CTTCACATGTGTCCTGCGATTACTTACGTACTAGTGGTGGTGGTGGTGGTGGGCAGACTCGAAGGTGAAGCTGGTGCCG |
| porTermUTR R | GCGGATAACAATTTCACACAGGAAACAGCTATGACCATGGCGCGCCGCCCAGCCTCTGGC |
| hphFnarI | AAAAAGGCGCCCCGTCGACAGAAGATGATATTGAAGGAGC |
| hphRnarI | AAAAAGGCGCCAGCTGACATCGACACCAACG |
| 5' Prom::pRS416 | GTAACGCCAGGGTTTTCCCAGTCACGACGTTGTAAAACGAATCGGGATTACAATGCAGGG |
| NcPprom:cDNA 3' | TGGCCGACTTGGCGATGTCAGAGAAAGCGGGAACAGCCATTGTGAAAGATGTTGTGTGAG |
