# Supplementary material for: A C-Terminally Truncated Variant of Neurospora crassa VDAC Assembles Into a Partially Functional Form in the Mitochondrial Outer Membrane and Forms Multimers in vitro
Source: Front Physiol. 2021 Sep 17;12:739001. doi: 10.3389/fphys.2021.739001 (PMC8485043; doi:10.3389/fphys.2021.739001)
Supplement: Supplementary file 4 [file Table_4.docx]

**Supplementary Table S4. CD Secondary Structure Deconvolution**

| Sample | α-Helix (%) | β-Strand (%) | β-Turn (%) | Other (%) | NRMSD |
| --- | --- | --- | --- | --- | --- |
| VDAC-ΔC Monomer | 6 | 52 | 9 | 33 | 0.037 |
| VDAC-ΔC Dimer | 6 | 50 | 10 | 34 | 0.035 |
| WT-VDAC* | 6 | 63 | 6 | 25 | 0.015 |

*****WT-VDAC values are taken from (Ferens et al., 2019)
