# Supplementary material for: A C-Terminally Truncated Variant of Neurospora crassa VDAC Assembles Into a Partially Functional Form in the Mitochondrial Outer Membrane and Forms Multimers in vitro
Source: Front Physiol. 2021 Sep 17;12:739001. doi: 10.3389/fphys.2021.739001 (PMC8485043; doi:10.3389/fphys.2021.739001)
Supplement: Supplementary file 5 [file Table_5.docx]

**Supplementary Table S5. SEC-MALS polydispersity values from ASTRA analysis**

| **Sample** | **Polydispersity (Mw/Mn)** |
| --- | --- |
| WT-VDAC | 1.000 |
| VDAC-ΔC-Monomer | 1.003 |
| VDAC-ΔC-Dimer | 1.001 |
| VDAC-ΔC-Tetramer | 1.000 |
