# Supplementary material for: A C-Terminally Truncated Variant of Neurospora crassa VDAC Assembles Into a Partially Functional Form in the Mitochondrial Outer Membrane and Forms Multimers in vitro
Source: Front Physiol. 2021 Sep 17;12:739001. doi: 10.3389/fphys.2021.739001 (PMC8485043; doi:10.3389/fphys.2021.739001)
Supplement: Supplementary file 6 [file Data_Sheet_1.DOCX]

**Supplementary Methods**

**Isolation of *N. crassa* mitochondria**

Mitochondria were isolated from wild-type (FGSC 9718) and cultures grown at 30^o^C for 16-18 hours. The differential centrifugation method described in (Harkness et al., 1994) was used for mitochondrial purification. In brief, *N. crassa* was grown in liquid Vogel’s media for 14-16 hours at 30^o^C. Mycelia were harvested by filtration, ground with sand in SEM buffer (250 mM sucrose, 1 mM EDTA, 9 mM 3-[N-Morpholino]-propanesulfonic acid (MOPS), pH 7.5). Cell debris and sand were removed by 2 rounds of centrifugation (3000*xg* for 5 minutes) and mitochondria were collected by centrifguation at 17000*xg* for 12 minutes). Mitochondrial pellets were resuspended in SEM and protein concentrations determined using the Bradford assay (Sigma, St. Louis, MO, USA).

**Western blot detection**

Equal amounts of mitochondrial protein (25 µg per lane) from each strain were separated through a 12% SDS-PAGE gel and then transferred to nitrocellulose. The resulting blots were probed with primary antibodies against residues 7-20 of VDAC, or purified Tom70; both antibodies were gifts from Drs. R. Lill, K. Hell and W. Neupert, Universität München. Secondary antibodies, coupled to horseradish peroxidase were purchased from Sigma-Aldrich (Oakville, ON). The Pierce™ ECL Western Blotting Substrate (Fisher Scientific, Ottawa, ON) was used and chemiluminescent signals were detected using an Alpha Innotech FluorChem 8900 imager.

**Purification of recombinant VDAC and VDAC-ΔC**.

The cDNA for VDAC-ΔC was cloned into pET21b, generating a coding sequence for VDAC-ΔC with the vector-encoded C-terminal his-tag. The clone was confirmed by DNA sequencing. The pET21b clone for full-length, C-terminally his-tagged VDAC. and protein purification methods were described in (Ferens et al., 2019). Briefly, the proteins were expressed from pET21b in *E. coli* c43 cells and collected from inclusion bodies. Following denaturation in 6M guanidine-HCl, the proteins were purified by Ni-NTA chromatography, and subsequently diluted into 20 mM Tris, pH 8.0, 300 mM NaCl containing 1% (w/v) decyl-maltoside (DM) and dialyzed against the same buffer without DM overnight. Samples were concentrated and folded protein was separated from aggregates by passage through a 24-mL capacity Superdex 200 increase column equilibrated with 20 mM MOPS pH 7.0, 100 mM NaCl, and 0.3% (w/v) DM.

**Circular Dichroism Spectrapolarimetry**

SEC Separated VDAC samples were diluted to 0.2 mg/ml with 20 mM MOPS pH 7.0, 100 mM NaCl in 0.3% (w/v) DM buffer. CD measurements were taken using a J-810 Circular Dichroism Spectropolarimeter (JASCO, Tokyo, Japan). Measurements were recorded from 190 nm to 260 nm with a 1 nm step, each curve represents the average of 3 technical replicate measurements minus the contribution of the buffer. Secondary structure deconvolution was done using the Dichroweb data processing server (<http://dichroweb.cryst.bbk.ac.uk/>) ((Lobley et al., 2002), (Whitmore and Wallace, 2004), (Whitmore and Wallace, 2008)) using the CDSSTR ((Compton and Johnson, 1986), (Manavalan and Johnson, 1987), (Sreerama and Woody, 2000)) algorithm and the SMP180 reference set ((Abdul-Gader et al., 2011)).

**References for Supplementary File 1**

Abdul-Gader, A., A.J. Miles, and B.A. Wallace. 2011. A reference dataset for the analyses of membrane protein secondary structures and transmembrane residues using circular dichroism spectroscopy. *Bioinformatics*. 27:1630-1636.

Compton, L.A., and W.C. Johnson, Jr. 1986. Analysis of protein circular dichroism spectra for secondary structure using a simple matrix multiplication. *Anal Biochem*. 155:155-167.

Ferens, F.G., T.R. Patel, G. Oriss, D.A. Court, and J. Stetefeld. 2019. A cholesterol analog induces an oligomeric reorganization of VDAC. *Biophys J*. 116:847-859.

Harkness, T.A., F.E. Nargang, I. van der Klei, W. Neupert, and R. Lill. 1994. A crucial role of the mitochondrial protein import receptor MOM19 for the biogenesis of mitochondria. *J Cell Biol*. 124:637-648.

Lobley, A., L. Whitmore, and B.A. Wallace. 2002. DICHROWEB: an interactive website for the analysis of protein secondary structure from circular dichroism spectra. *Bioinformatics*. 18:211-212.

Manavalan, P., and W.C. Johnson, Jr. 1987. Variable selection method improves the prediction of protein secondary structure from circular dichroism spectra. *Anal Biochem*. 167:76-85.

Sreerama, N., and R.W. Woody. 2000. Estimation of protein secondary structure from circular dichroism spectra: comparison of CONTIN, SELCON, and CDSSTR methods with an expanded reference set. *Anal Biochem*. 287:252-260.

Whitmore, L., and B.A. Wallace. 2004. DICHROWEB, an online server for protein secondary structure analyses from circular dichroism spectroscopic data. *Nucleic Acids Res*. 32:W668-673.

Whitmore, L., and B.A. Wallace. 2008. Protein secondary structure analyses from circular dichroism spectroscopy: methods and reference databases. *Biopolymers*. 89:392-400.
