# Supplementary figures and images for: A C-Terminally Truncated Variant of Neurospora crassa VDAC Assembles Into a Partially Functional Form in the Mitochondrial Outer Membrane and Forms Multimers in vitro
Source: Front Physiol. 2021 Sep 17;12:739001. doi: 10.3389/fphys.2021.739001 (PMC8485043; doi:10.3389/fphys.2021.739001)

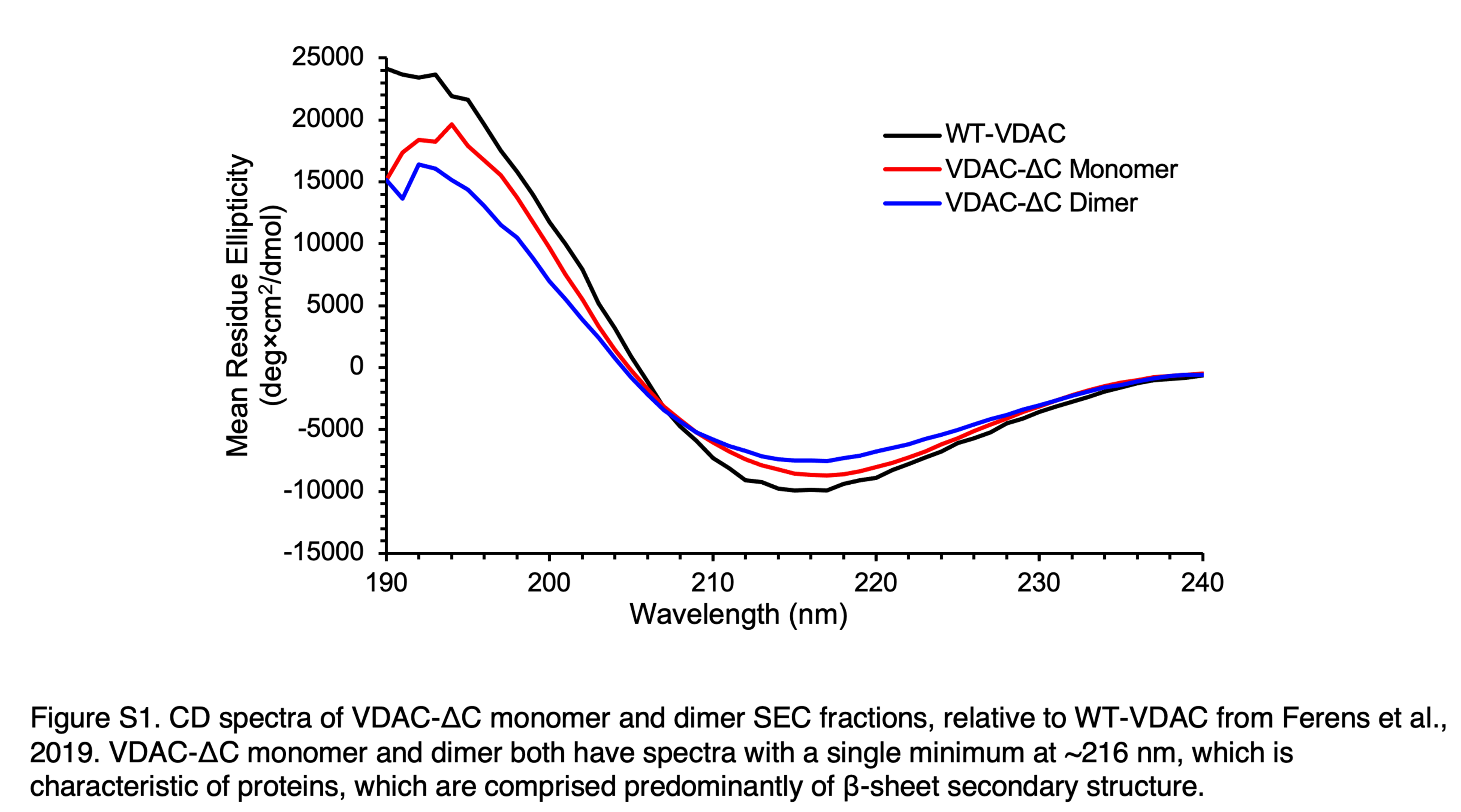

Supplement: Supplementary file 7 [file Image_1.TIFF]

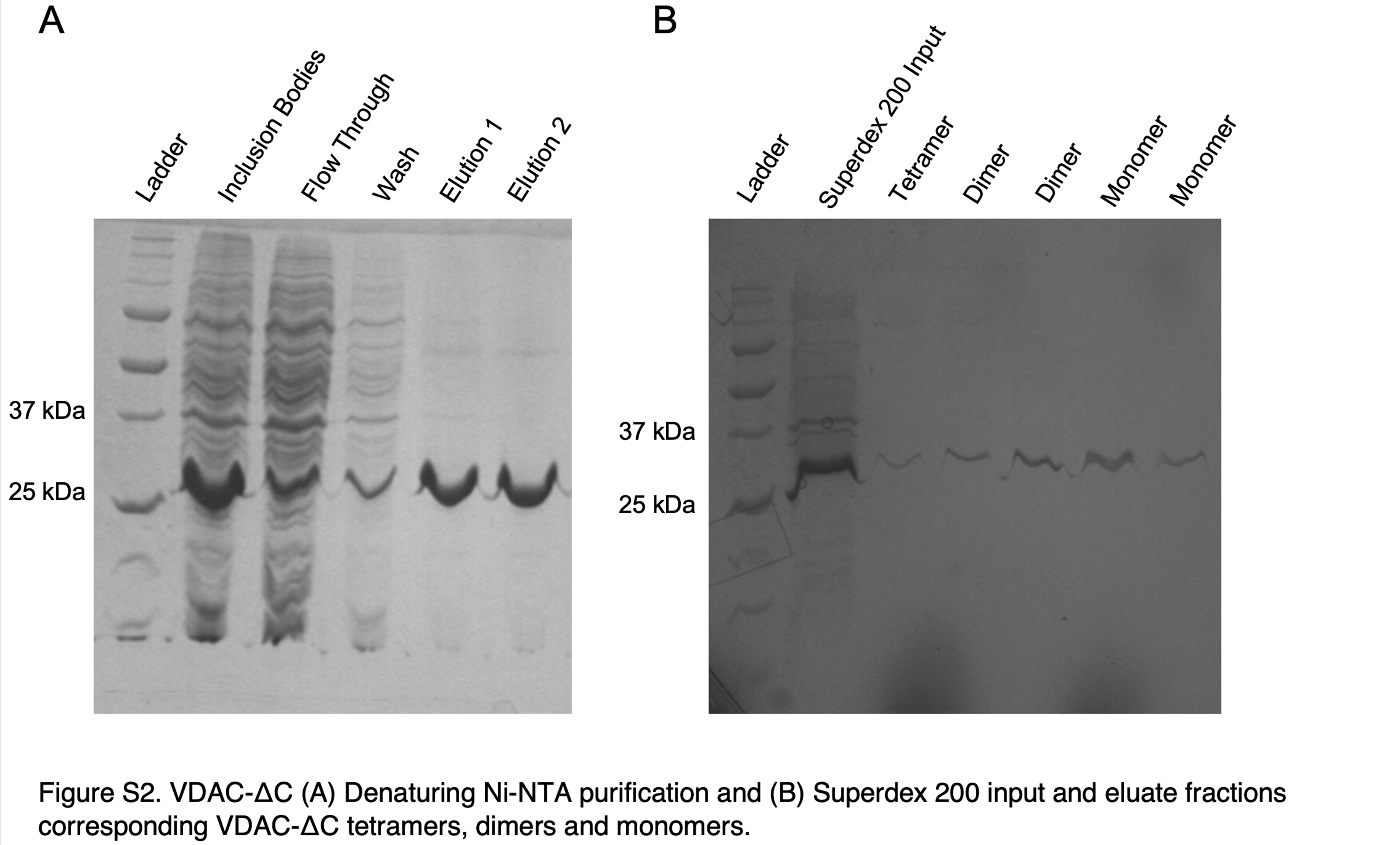

Supplement: Supplementary file 8 [file Image_2.TIFF]
